# The complete mitochondrial genome of *Schisandra sphenanthera* (Schisandraceae)

**DOI:** 10.1080/23802359.2018.1532346

**Published:** 2018-10-30

**Authors:** Xianxian Yu, Yanlei Feng, Wei Zhai, Min Chen, Guoxi Wu

**Affiliations:** a School of Urban–Rural Planning and Landscape Architecture, Xuchang University, Xuchang, China;; b Institute of Evolution and Biodiversity, University of Muenster, Muenster, Germany;; c College of Life Sciences, Shaanxi Normal University, Xi’an, China;; d Institute of Botany, Jiangsu Province and Chinese Academy of Sciences, Nanjing, China

**Keywords:** *Schisandra sphenanthera*, mitochondrial genome, phylogeny

## Abstract

*Schisandra sphenanthera* (Austrobaileyales) is a famous traditional Chinese medicine being long-history used, is also one of early-diverging angiosperms and important links to uncover the early evolution of angiosperms. Here the complete mitochondrial genome of *S. sphenanthera* was obtained for the first time. It is 1,106,521 bp in length with 46.4% GC content. It contains 58 genes, including 41 protein coding genes, three ribosomal RNA genes and 14 transfer RNA genes. Phylogenetic analysis indicated that *S. sphenanthera* was placed in the basal angiosperm just after *Amborella* and *Nuphar*. The mitogenome of *S. sphenanthera* would provide a reliable genetic and evolutionary resource.

*Schisandra sphenanthera* Rehd. et E. H. Wilson, is a kind of liana and a horticultural plant with edible fruit in Austrobaileyales. Austrobaileyales is an early-diverging extant angiosperm branch just following Amborellales and Nymphaeales, thus provide an important link to uncover the origin and early evolution of angiosperms (APG IV 2016). Species in *Schisandra* are mainly native to east and southeast Asia but one species *S. glabra* in North America (Xia et al. 2009). Pharmacological studies on animals have shown that *Schizandra* affords a stress-protective effect against a broad spectrum of harmful factors (Iwata et al. 2004; Xiao et al. 2005; Panossian and Wikman 2008; Zhao et al. 2014; Jackson et al. 2017). Here we reported the complete mitochondrial genome (mitogenome) sequences of *S. sphenanthera* in Austrobaileyales for the first time. This would be helpful for the pharmaceutical, phylogenetic and mitochondrial evolutionary researches.

The whole genomic DNA was extracted from leaves of a mature *S. sphenanthera* plant which was growing in the natural forest habitat of Baoji city, Shaanxi province, China (34°05.313N107°42.315E). Voucher specimen and DNA sample (CHEN M. 20130709) were deposited in the herbarium of Institute of Botany, CAS (PE). Total 4.05 G 150 bp paired end raw reads using Illumina HiSeq2500 (Illumina, San Diego, CA) were generated and then trimmed low quality bases (<Q20) using NGS QC Toolkit (Patel and Jain 2012). Around 3.7 G trimmed reads were used to assemble mitogenome by Velvet v1.2.10 (Zerbino and Birney 2008). Contigs were connected manually in Geneious R8 (Biomatters, Inc., Auckland, New Zealand). Genome was annotated based on the former published mitogenomes of *Liriodendron tulipifera* (GenBank: NC_021152) and *Nymphaea colorata* (NC_037468). Plastid-derived DNAs were determined by searching against *Schisandra chinensis* chloroplast genome (NC_034908) with Blastn v2.4.0 (Camacho et al. 2009) and only fragments >200 bp were considered. Dispersed repeats were searched in Geneious with Repeat Finder v1.0 plugin, with settings minimum length 100 bp and maximum mismatch 5%.

**Figure 1.**
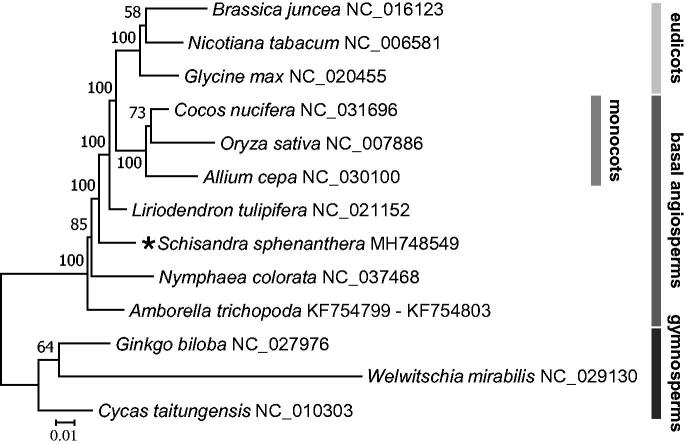
Phylogenetic tree based on 13 complete mitogenome sequences. The tree was constructed using RAxML method and bootstrap support value from 1000 replicates are shown above branches. All the mitogenome sequences are available in GenBank, the accession numbers are listed right to their scientific names.

The mitogenome of *S. sphenanthera* is 1,106,521 bp in length with 46.4% GC content and under the accession number MH748549. The mitogenome contains 58 genes, including 41 protein coding genes, three ribosomal RNA genes and 14 transfer RNA genes. Ten genes have introns and three of them are trans-spliced. Total plastid-derived DNA length is 26 kb, around 2.3% of the genome. Forty-six repeat pairs were found and nine of them are >500 bp. The total repeat length is around 74 kb.

A total of 41 mitochondrial coding genes of *S. sphenanthera* and another 12 seed plants were aligned with Mafft v7.306b (Yamada et al. 2016). The maximum likelihood tree was performed by using RAxML v8.2.10 (Stamatakis 2014) with 1000 bootstrap iterations in CIPRES website (Miller et al. 2010). The tree showed that *S. sphenanthera* places basal angiosperm branches just after *Amborella* (Amborellales) and *Nuphar* (Nymphaeales), which was consistent with the results by Angiosperm Phylogeny Group IV (APG IV 2016) (Figure 1). The complete mitogenome of *S. sphenanthera* would represent a useful genetic resource to the further biological and evolutionary studies.
